# The value of ACTN1 in the diagnosis of cutaneous squamous cell carcinoma: A continuation study

**DOI:** 10.1111/srt.13252

**Published:** 2023-03-29

**Authors:** Yuan Tao, Lu Xiao‐hong, Ke Guo‐lin, Hu hua, Jiang Jia‐hui, Ci Chao

**Affiliations:** ^1^ Department of Dermatology Yijishan Hospital the First Affiliated Hospital of Wannan Medical College Wuhu China; ^2^ Department of Dermatology The First Affiliated Hospital of Xinxiang Medical University Weihui China

**Keywords:** ACTN1, biomarkers, Bowen's disease, cutaneous squamous cell carcinoma

To The Editor,

1

Cutaneous squamous cell carcinoma (CSCC) originates from epidermal keratinocytes or adnexal structures, including eccrine glands and (or) pilosebaceous units. CSCC is the second most common nonmelanoma skin cancer, and its incidence has increased in recent years. According to several studies reported, about 15–35 per 100 000 individuals are diagnosed with CSCC each year, and the incidence of CSCC is expected to increase by 2%−4% per year.^1^ On the other hand, the incidence of primary CSCC has increased by 50%−300% globally, especially among Caucasian populations in New Zealand, Australia, and North America over the last 3 decades.^2^ Although the early‐stage CSCC usually has a good prognosis, the metastatic CSCC can be difficult to treat. Bowen's disease is also known as squamous cell carcinoma in situ. It is estimated that in general population around 3%–5% of Bowen's disease transform into invasive CSCC.^3^ In recent years, a variety of studies have been devoted to exploring the molecular mechanism and target proteins of the progression of in situ carcinoma to invasive CSCC via omics methods.^4^, ^5^, ^6^ Besides, the lesions appearance of the Bowen's disease and CSCC were similar and not indistinguishable. Therefore, the histopathological examination results, the only gold standard for evaluating Bowen's disease progression to CSCC. In addition, early assessment of Bowen's disease progression to CSCC is particularly important for treatment methods options. According to our previous investigated, when compared with lesions of Bowen's disease, the Tenascin C (TNC), FSCN1, SERPINB1, ACTN1, and RAB31 in CSCC tissues were significantly upregulated, while COL3A1, COL1A1, and CD36 were significantly downregulated.^6^ However, the serum levels of these proteins in CSCC was unclear, and in the present study, we explored the serum levels of these proteins differences in CSCC relative to Bowen's disease. We also discussed the diagnostic value of these proteins expression in CSCC.

In this study, a total of 55 patients were diagnosed with CSCC (CSCC group) in the Department of Dermatology, Yijishan Hospital and the First Affiliated Hospital of Xinxiang Medical University between January 2020 and January 2022 were enrolled. All patients were diagnosed with CSCC according to histopathological examination findings. The patients with CSCC comprised 27 males (49.09%) and 28 females (50.91%) aged between 39 and 69 years, with an average age of 52.29 ± 5.88 years. The CSCC patients with first diagnosed and never treated with any biological agents such as programmed cell death protein 1. On the other hand, 40 patients with Bowen's disease (BD group) and 30 healthy volunteers (Control group) were also included in this study: These 70 individuals, with no history of severe infections and malignant tumors, in the same period were enrolled. The BD group comprised 22 males (55.00%) and 18 females (45.00%) aged between 44 and 61 years, with an average age of 53.55 ± 4.56 years. The control group comprised 16 males (53.33%) and 14 females (46.67%) aged between 41 and 66 years, with an average age of 50.30 ± 6.39 years. There was no significant difference in gender and age among the three groups (*p* > 0.05). The clinical baseline characteristics of three groups are shown in Table S1. All individuals signed the informed consent. The serum levels of TNC (Abcam), FSCN1 (AssayGenie), SERPINB1 (AssayGenie), ACTN1 (AssayGenie), RAB31 (Abbexa), COL3A1 (AssayGenie), COL1A1 (AssayGenie), and CD3 (AssayGenie) were measured by an enzyme‐linked immuno sorbent assay kit. The minimum‐to‐maximum detection limits were (93.7∼6000) pg/ml for TNC, (0.78∼50) ng/ml for FSCN1, (0.313∼20) ng/ml for SERPINB1, (0.156∼10) ng/ml for ACTN1, (0.156∼10) ng/ml for RAB31, (31.25∼2000) ng/ml for COL3A1, (0.313∼20) ng/ml for COL1A1, and (0.156∼10) ng/ml for CD36. On the other hand, the transcriptomic data of CSCC (GSE45216) were selected and downloaded from the GEO database (https://www.ncbi.nlm.nih.gov/pmc/). In GSE45216, a total of 30 samples with CSCC (GSM1099226∼GSM1099255) and 10 samples with Actinic keratosis (GSM1099256∼GSM1099265) were selected and enrolled. Actinic keratosis, similar with Bowen's disease, is a common premalignant skin lesion characterized by itraepithelial keratinocyte dysplasia and molecular alterations shared with CSCC. Clinically, the majority of patients with AK will evolve into CSCC.^7^ The RNA was extracted by laser capture microdissection from 10 Actinic keratosis and 30 CSCC, for analysis using the Affymetrix HG U133 Plus 2.0 microarrays. The transcriptomic data were calculated using GEO2R online (https://www.ncbi.nlm.nih.gov/geo/geo2r/). The differentially expressed transcriptomic data between CSCC and AK were further identified using a log_2_FC > 1/ ← 1 and adjusted *p* values < 0.05. Importantly, the GSE45216 was used to validate the target protein expression value difference between CSCC and AK.

According to our results, we only found the expression levels of SERPINB1 and ACTN1 in serum of patients with CSCC were significantly higher than that of Bowen's disease (*p* < 0.05). The data were presented in Figure S1. Furthermore, the results were furthered validation in GSE45216, and we confirmed that the ACTN1 expression value in CSCC was significantly higher than that of Actinic keratosis (*p* < 0.05). The data are presented in Figure 1. Combined with the above results, we proved that the ACTN1 was a biomarker protein of the progression of in situ carcinoma to invasive CSCC. On the other hand, the receiver operating characteristic curve was drawn to analyze the efficacy of the serum ACTN1 expression value in the diagnosis of CSCC. The areas under the curve of ACTN1 for the diagnosis of CSCC were 0.841. The sensitivity and specificity were 86.40% and 82.50%, respectively. The data are presented in Figure S2. The association between ACTN1 expression level and clinicopathologic features in patients with CSCC was analyzed. The serum expression level of ACTN1 was related to the primary tumor diameter, tumor cell differentiation degree and invasion depth (*p* < 0.05). The results are showed in Table 1.

CSCC in situ is also known as Bowen's disease. If left untreated, Bowen's disease may progress to invasive CSCC with the incidence of 3%–5%.^8^ The exact mechanisms by which Bowen's disease progresses to CSCC are complicated and merits more attention. ACTN1, an actin cross‐linking protein, it is involved in the tumorigenesis and development of certain cancers. Previous study indicated that the ACTN1 regulates the epithelial‐mesenchymal transition and tumorigenesis of gastric cancer via the AKT/GSK3 β/β‐catenin pathway.^9^ Besides, the ACTN1 is also over‐expressed in hepatocellular carcinoma tissues by suppressing Hippo signaling via physical interaction with MOB1.^10^ On the other hand, higher ACTN1 protein levels were significantly associated with poor prognosis in Oral Squamous Cell Carcinoma.^11^ Therefore, our results suggested that ACTN1 acts as tumor promoter and also serve as a diagnostic biomarker of Bowen's disease progression to CSCC. However, the exact mechanism of ACTN1 regulates CSCC progression still needs to be elucidated.

**FIGURE 1:**
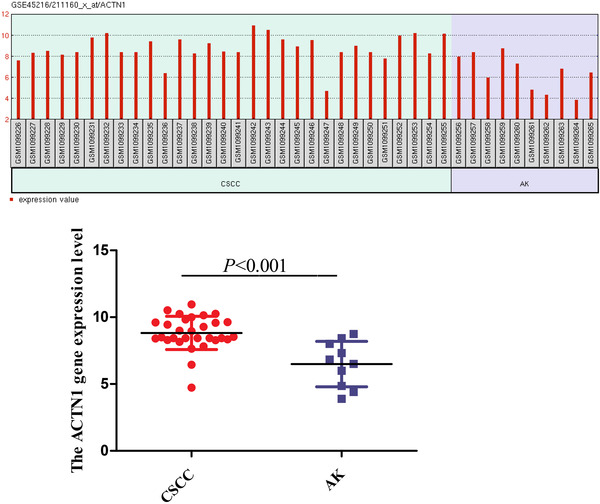
The ACTN1 gene expression level between CSCC and AK from GSE45216

**TABLE 1:** The association between ACTN1 expression levels and clinic‐pathological features in

Cutaneous squamous cell carcinoma (CSCC) (*n* = 55)				
Clinic‐pathological factors	Cases ACTN1	Expression levels	Statistics value	*p‐*Value
Age, years			1.162	0.251
≥55	17	7.52 ± 1.22		
<55	38	6.98 ± 1.73		
Gender			1.846	0.070
Male	27	6.70 ± 1.71		
Female	28	7.47 ± 1.37		
Sun exposure			0.667	0.507
Yes	29	7.28 ± 1.74		
No	26	6.99 ± 1.45		
Primary tumor diameter, cm			5.414	<0.001
≥3	30	8.01 ± 1.24		
<3	25	6.11 ± 1.36		
Tumor cell differentiation			3.627	<0.001
Well	22	6.28 ± 1.89		
Moderate‐poor	33	7.72 ± 1.05		
Invasion depth			4.163	<0.001
Dermis/subcutaneous fat	16	5.92 ± 1.74		
Bone/muscle/Perineural	39	7.65 ± 1.24		

## CONFLICT OF INTEREST

The authors declare that they have no competing interests.

2

## ETHICS STATEMENT

All study participants signed an informed consent form prior to participation. All authors gave their consent for publication.

## Supporting information

Supporting Information

Supporting Information

Supporting Information

## Data Availability

All the data for this study will be made available upon reasonable request to the corresponding author.

## References

[1] Rickert RR , Brodkin RH , Hutter RV . Bowen's disease. CA Cancer J Clin. 1977;27(3):160–166. 406015 10.3322/canjclin.27.3.160

[2] Brougham ND , Tan ST . The incidence and risk factors of metastasis for cutaneous squamous cell carcinoma–implications on the T‐classification system. J Surg Oncol. 2014;110(7):876–882. 25088537 10.1002/jso.23731

[3] Burton KA , Ashack KA , Khachemoune A . Cutaneous squamous cell carcinoma: a review of high‐risk and metastatic disease. Am J Clin Dermatol. 2016;17(5):491–508. 27358187 10.1007/s40257-016-0207-3

[4] Azimi A , Lo K , Kim J , et al. Investigating proteome changes between primary and metastatic cutaneous squamous cell carcinoma using SWATH mass spectrometry. J Dermatol Sci. 2020;99(2):119–127. 32651104 10.1016/j.jdermsci.2020.06.012

[5] Azimi A , Yang P , Ali M , et al. Data independent acquisition proteomic analysis can discriminate between actinic keratosis, Bowen's disease, and cutaneous squamous cell carcinoma. J Invest Dermatol. 2020;140(1):212–222.e11. 10.1016/j.jid.2019.06.128 31254517

[6] Biao T , Cai‐Feng H , Xiao‐Hong L , et al. From Bowen disease to cutaneous squamous cell carcinoma: eight markers were verified from transcriptomic and proteomic analyses. J Transl Med. 2022;20(1):416. 10.1186/s12967-022-03622-1 36085041 PMC9462620

[7] Navarrete‐Dechent C , Marghoob AA , Marchetti MA . Contemporary management of actinic keratosis. J Dermatolog Treat. 2021;32(5):572–574. 31621454 10.1080/09546634.2019.1682504

[8] Morton CA , Birnie AJ , Eedy DJ . British Association of Dermatologists' guidelines for the management of squamous cell carcinoma in situ (Bowen's disease) 2014. Br J Dermatol. 2014;170(2):245–260. 24313974 10.1111/bjd.12766

[9] Zhang S , Wang J , Chen T , et al. α‐Actinin1 promotes tumorigenesis and epithelial‐mesenchymal transition of gastric cancer via the AKT/GSK3 β/β‐catenin pathway. Bioengineered. 2021;12(1):5688–5704. 34546849 10.1080/21655979.2021.1967713 PMC8806412

[10] Chen Q , Zhou XW , Zhang AJ , et al. ACTN1 supports tumor growth by inhibiting Hippo signaling in hepatocellular carcinoma. J Exp Clin Cancer Res. 2021;40(1):23. 10.1186/s13046-020-01821-6 33413564 PMC7791991

[11] Xie GF , Zhao LD , Chen Q , et al. High ACTN1 is associated with poor prognosis, and ACTN1 silencing suppresses cell proliferation and metastasis in oral squamous cell carcinoma. Drug Des Devel Ther. 2020;14:1717–1727. 10.2147/DDDT.S244516 PMC7211328 32440097

