# Supplementary material for: The value of ACTN1 in the diagnosis of cutaneous squamous cell carcinoma: A continuation study
Source: Skin Res Technol. 2023 Mar 29;29(4):e13252. doi: 10.1111/srt.13252 (PMC10234166; doi:10.1111/srt.13252)
Supplement: Supplementary file 1 — Supporting Information [file SRT-29-e13252-s002.pdf]

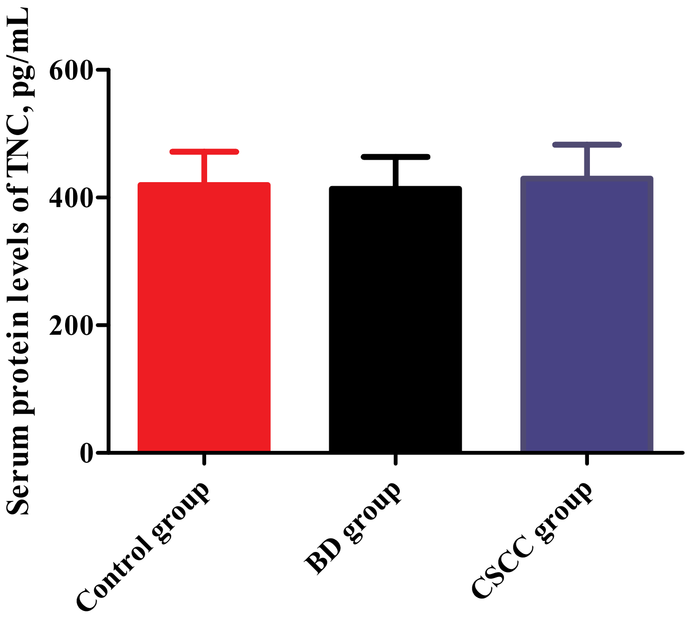
Serum protein levels of TNC, pg/mL
600
400
200
0
Control group
BD group
CSCC group

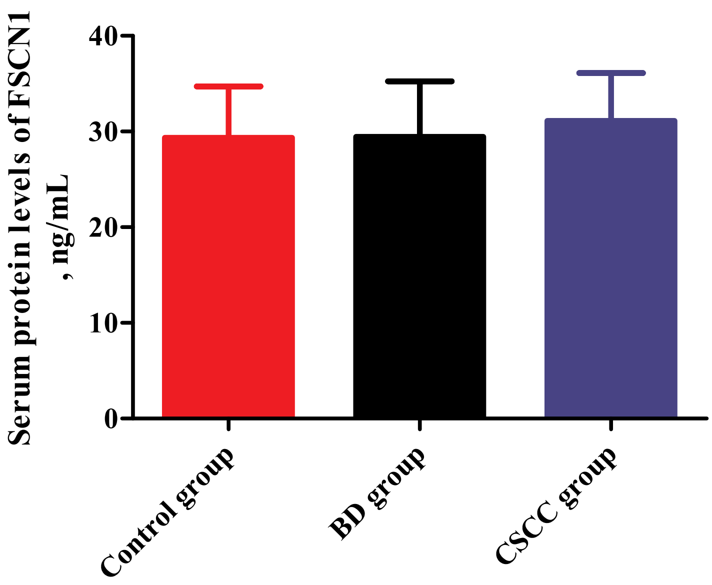
Serum protein levels of FSCN1 , ng/mL
40
30
20
10
0
Control group
BD group
CSCC group

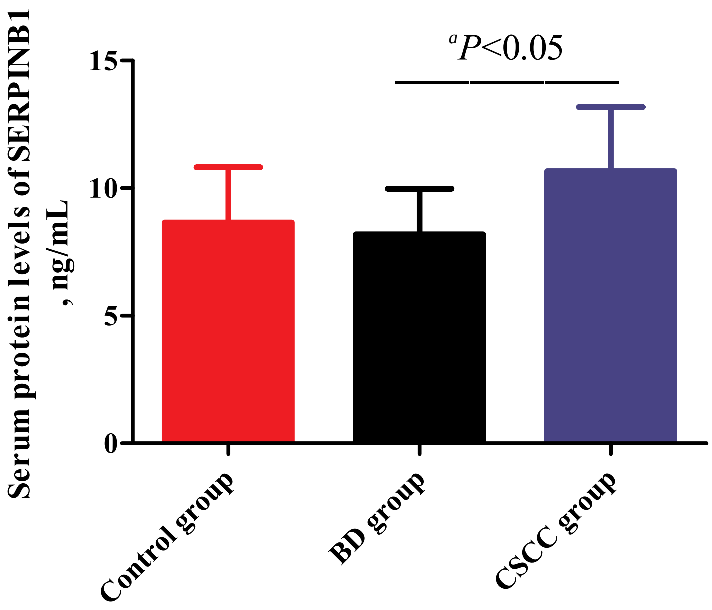

aP<0.05
Serum protein levels of SERPINB1, ng/mL
15
10
5
0
Control group
BD group
CSCC group

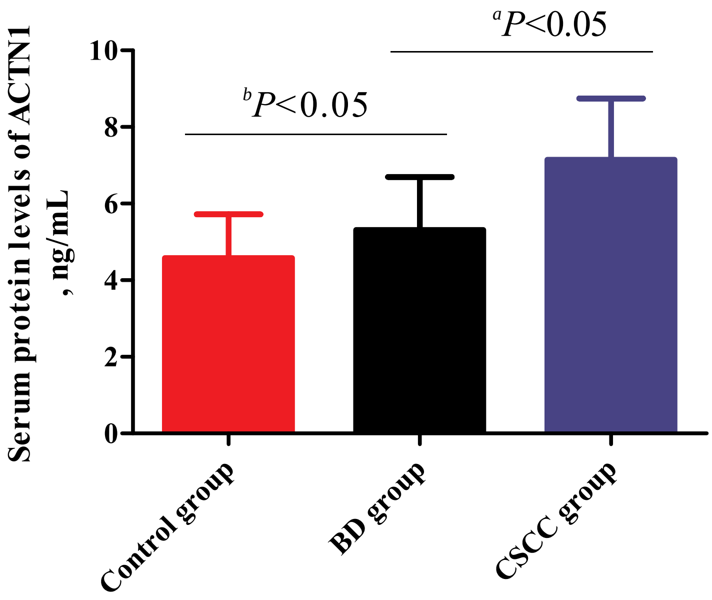

Serum protein levels of ACTN1, ng/mL
10
8
6
4
2
0
bP<0.05
aP<0.05
Control group
BD group
CSCC group

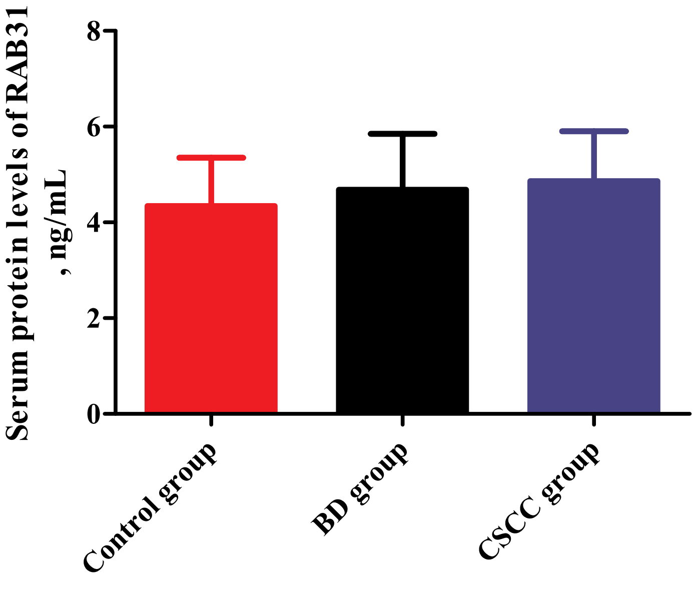

Serum protein levels of RAB31
, ng/mL
8
6
4
2
0
Control group
BD group
CSCC group

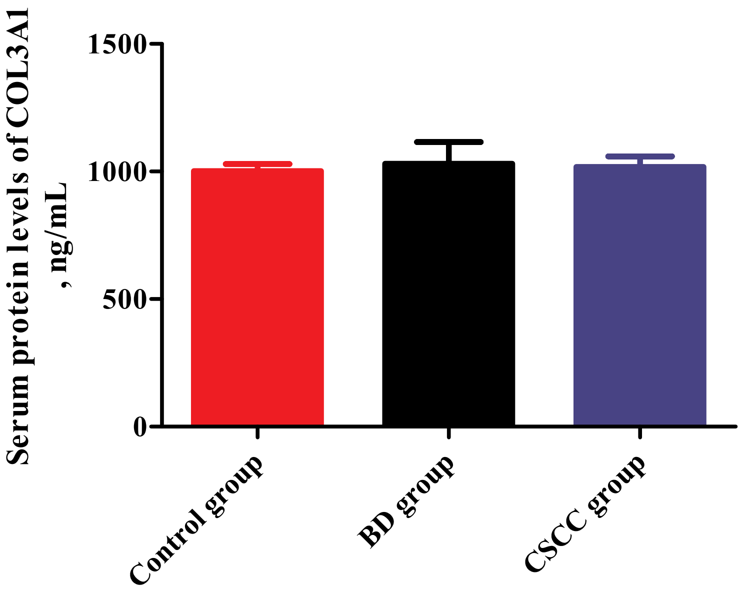

Serum protein levels of COL3A1
, ng/mL
1500
1000
500
0
Control group
BD group
CSCC group

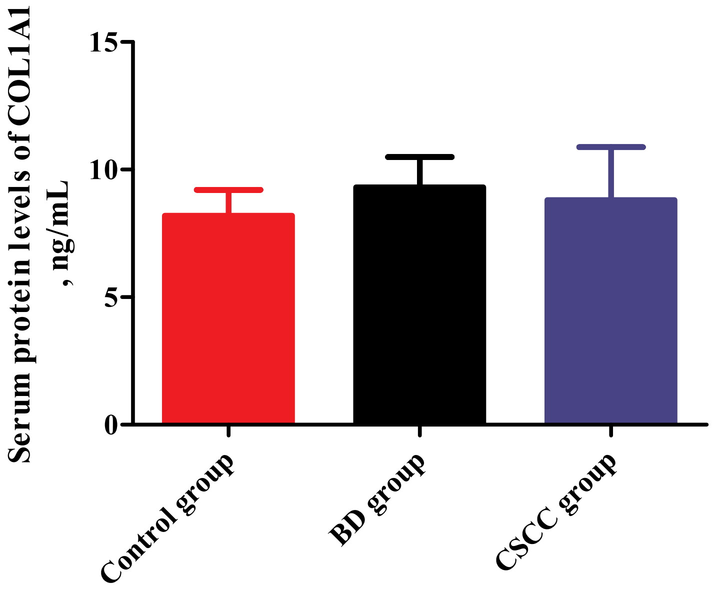
Serum protein levels of COL1A1
, ng/mL
15
10
5
0
Control group
BD group
CSCC group

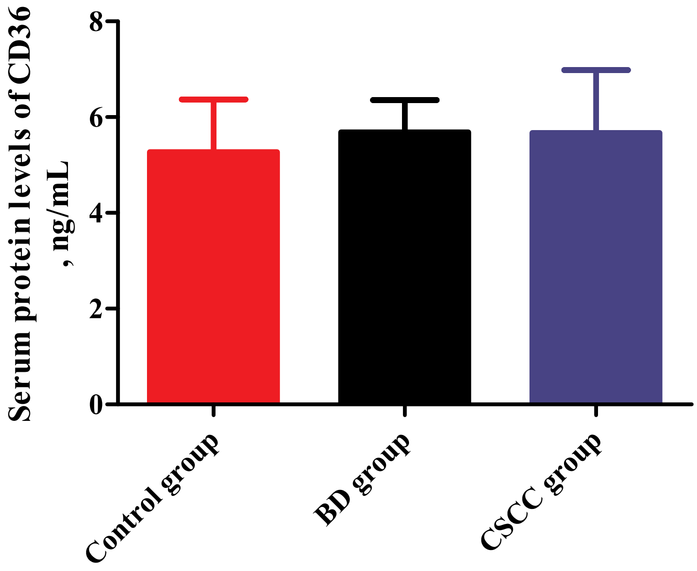

Supplemental Figure 1. Serum protein levels of TNC, FSCN1, SERPINB1, ACTN1, RAB31, COL3A1, COL1A1 and CD36 between three groups.
