# Supplementary material for: The value of ACTN1 in the diagnosis of cutaneous squamous cell carcinoma: A continuation study
Source: Skin Res Technol. 2023 Mar 29;29(4):e13252. doi: 10.1111/srt.13252 (PMC10234166; doi:10.1111/srt.13252)
Supplement: Supplementary file 2 — Supporting Information [file SRT-29-e13252-s001.pdf]

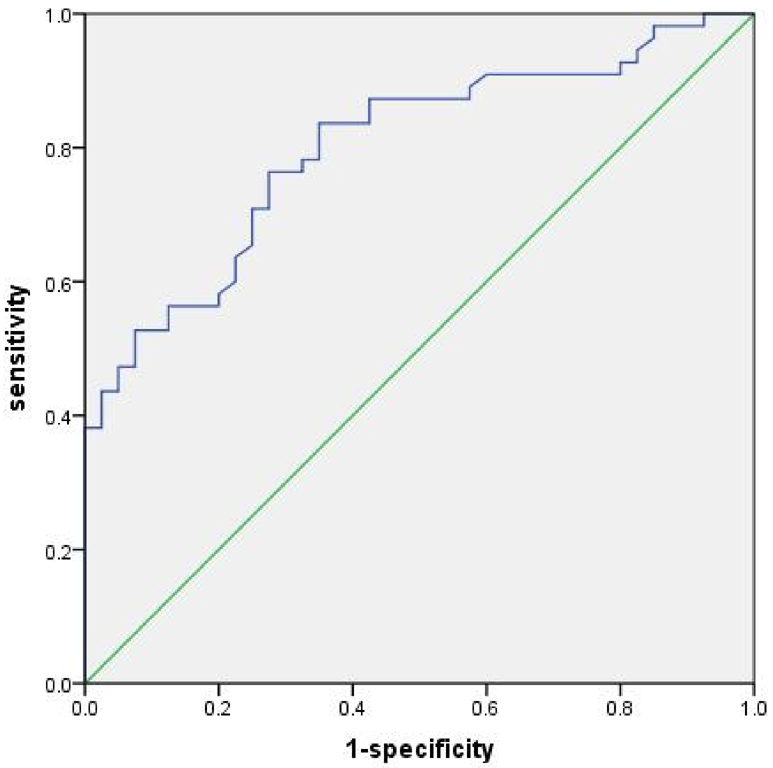

Supplemental Figure 2. The ROC curve of ACTN1 protein expression level was used to distinguish between CSCC and Bowen disease.
