# Supplementary material for: The value of ACTN1 in the diagnosis of cutaneous squamous cell carcinoma: A continuation study
Source: Skin Res Technol. 2023 Mar 29;29(4):e13252. doi: 10.1111/srt.13252 (PMC10234166; doi:10.1111/srt.13252)
Supplement: Supplementary file 3 — Supporting Information [file SRT-29-e13252-s003.pdf]

Supplemental Table 1. The comparison of clinical baseline characteristics of three groups.

Groups	Gender, male	Age, years
Control group (n=30)	16, 53.33%	50.30±6.39
BD group (n=40)	22, 55.00%	53.55±4.56
CSCC group (n=55)	27, 49.09%	52.29±5.88
Statistics value	0.197	2.868
P value	0.658	0.061
